# Prognostic Prediction of Cytogenetically Normal Acute Myeloid Leukemia Based on a Gene Expression Model

**DOI:** 10.3389/fonc.2021.659201

**Published:** 2021-05-27

**Authors:** Liu Yang, Houyu Zhang, Xue Yang, Ting Lu, Shihui Ma, Hui Cheng, Kuangyu Yen, Tao Cheng

**Affiliations:** ^1^ State Key Laboratory of Experimental Hematology, National Clinical Research Center for Blood Diseases, Institute of Hematology & Blood Diseases Hospital, Chinese Academy of Medical Sciences & Peking Union Medical College, Tianjin, China; ^2^ Department of Stem Cell and Regenerative Medicine, Peking Union Medical College, Tianjin, China; ^3^ Center for Stem Cell Medicine, Chinese Academy of Medical Sciences, Tianjin, China; ^4^ School of Biology and Biological Engineering, South China University of Technology, Guangzhou, China

**Keywords:** cytogenetically normal acute myeloid leukemia, prognosis, biomarker, immune dysfunction, bone marrow

## Abstract

Acute myeloid leukemia (AML) refers to a heterogeneous group of hematopoietic malignancies. The well-known European Leukemia Network (ELN) stratifies AML patients into three risk groups, based primarily on the detection of cytogenetic abnormalities. However, the prognosis of cytogenetically normal AML (CN-AML), which is the largest AML subset, can be hard to define. Moreover, the clinical outcomes associated with this subgroup are diverse. In this study, using transcriptome profiles collected from CN-AML patients in the BeatAML cohort, we constructed a robust prognostic Cox model named NEST (Nine-gEne SignaTure). The validity of NEST was confirmed in four external independent cohorts. Moreover, the risk score predicted by the NEST model remained an independent prognostic factor in multivariate analyses. Further analysis revealed that the NEST model was suitable for bone marrow mononuclear cell (BMMC) samples but not peripheral blood mononuclear cell (PBMC) samples, which indirectly indicated subtle differences between BMMCs and PBMCs. Our data demonstrated the robustness and accuracy of the NEST model and implied the importance of the immune dysfunction in the leukemogenesis that occurs in CN-AML, which shed new light on the further exploration of molecular mechanisms and treatment guidance for CN-AML.

## Introduction

Acute myeloid leukemia (AML) is a heterogeneous group of hematopoietic disorders with diverse clinical outcomes (1). The initial recognition of this heterogeneity depends primarily on morphology (2). The French-American-British (FAB) Cooperative Group developed a classification system based on morphologic and cytochemical characteristics, which classified AML into eight subgroups (M0-M7) (3, 4). However, this classification provides limited prognostic guidance for AML patients (5).

Advances in sequencing technologies have contributed to an increased understanding of AML biology. Based on genetic abnormalities, the European Leukemia Network (ELN) risk stratification system classifies AML patients into three risk groups: favorable, intermediate, and adverse (**Table S1**) (6). The cytogenetic abnormalities associated with AML are recognized as being the most valuable prognostic factors (7). However, cytogenetically normal AML (CN-AML) represents the largest AML subset, comprising 45%–60% of all cases (8, 9). The prognosis of CN-AML must be assessed basing on genetic mutations alone due to the presentation of normal cytogenetic features (**Table S1**). In addition, the clinical outcomes of patients in this subgroup are also diverse and challenging to define (10).

According to the ELN recommendations, six genetic mutations have been demonstrated to be of prognostic significance among all AML patients, including mutations in *FLT3*, *NPM1*, *CEBPA*, *RUNX1*, *TP53*, and *ASXL1* (11). *NPM1* mutations occur at a high frequency, ranging from 25% to 35% of all AML patients and from 45.7% to 63.8% of all CN-AML patients (9). *FLT3* mutations were identified in approximately 20% of AML and 28%–34% of CN-AML patients (12). Aside from mutations in *NPM1* and *FLT3*, the mutation frequency of other genes in CN-AML is relatively low (6). Therefore, genetic mutations alone appear to be insufficient to provide a comprehensive prognostic assessment of CN-AML.

Genetic mutations can result in either the loss or gain of function and can subsequently influence the expression profiles of downstream genes. Given the diversity and uncertainty of prognoses among CN-AML patients, novel molecular markers may be discovered through the performance of transcriptome analyses that can be used to refine the risk stratification strategy for CN-AML patients. In recent decades, studies have identified that the expression of certain genes was correlated with poor prognosis in CN-AML (13–16). However, these studies have been associated with various limitations. For example, the identified prognostic factors have lacked consistency among different cohorts. And sample origins have been ignored when PBMCs and BMMCs were always mixed for analyses, whereas some studies have indicated that the proportions and properties differ between PBMCs and BMMCs (17, 18).

In this study, we integrated multiple transcriptome datasets [BeatAML (19), GSE71014 (20), GSE12417 (21), GSE6891 (22), TARGET-AML (23), and TCGA-LAML (11)] and identified nine prognostic markers in CN-AML BMMCs. We fitted a multivariate Cox proportional hazards model and developed a 9-gene model, named NEST (Nine-gEne SignaTure). The NEST model was able to provide a personalized prognostic value for risk assessment in CN-AML patients. Notably, our study suggested that the NEST model was applicable to BMMCs but not to PBMCs, which implied subtle differences between PBMCs and BMMCs in CN-AML patients. Our results pave the way for further explorations of the molecular mechanisms and prognostic markers associated with CN-AML.

## Materials and Methods

### Data Source and Preprocessing

We downloaded gene expression profiles (raw count) and clinical information of *de novo* CN-AML patients from the BeatAML cohort (http://www.vizome.org/aml) as a training dataset. The cohort includes samples from both bone marrow and peripheral blood. On the one hand, bone marrow samples were derived from 105 patients with *de novo* CN-AML and 21 healthy donors. There were 33 samples in total derived from healthy donors. Among them, 19 samples were BMMCs, and the remaining 14 samples were CD34^+^ bone marrow (CD34^+^) cells. Notably, all CD34^+^ cells were collected from three patients. CD34^+^ sample from one patient was included in each sequencing batch (for a total of 12 times sequencing this control RNA). On the other hand, peripheral blood samples included 43 patients with *de novo* CN-AML in BeatAML. Moreover, to validate our model, we selected bone marrow data from four external validation datasets of CN-AML. Of these, GSE71014 (n = 104) (20), GSE12417 (n = 73) (21) and GSE6891 (n = 88) (22) were microarray datasets downloaded from the GEO database (http://www.ncbi.nlm.nih.gov/geo/), and TARGET-AML (23) were gene expression profiles (https://ocg.cancer.gov/programs/target). Apart from these datasets, we also download the TCGA-LAML (11) dataset obtained from the TCGA data portal (https://gdc-portal.nci.nih.gov/). The sample origin of the TCGA-LAML was PBMCs. Due to the different treatment regimens and favorable outcomes of AML-M3 patients, we excluded them from all cohorts. Ensemble IDs from the BeatAML dataset were converted to gene symbol with a GTF file (Homo_sapiens.GRCh37.75.gtf) downloaded from GENCODE (https://www.gencodegenes.org/). For microarray datasets, the median value was regarded as the gene’s expression value for multiple probe sets corresponding to the same gene. The overall survival time and genetic mutation information were obtained from publications and the GEO database. No specific ethical approval is required for this study, as all datasets used were publicly available.

### Identification of Differentially Expressed Genes

The raw gene expression of the BeatAML dataset was normalized by the trimmed mean of M values (TMM) method with the “edgeR” package in the R platform (24). The voom method estimated the mean-variance relationship of the normalized data, generated a precision weight for each observation and entered the “limma” empirical Bayes analysis (25). Differences in gene expression with an adjusted P-value < 0.01 and absolute log2 fold change (log2FC) >= 2 were considered significant differences.

### Functional Enrichment Analysis

We used the “clusterProfiler” R package to perform Gene Ontology (GO) enrichment analysis (26). Moreover, DEGs were uploaded into the Ingenuity Pathway Analysis (IPA) system for core analysis (27). The ingenuity knowledge base (genes only) was selected as the reference set. IPA was performed to identify the canonical pathways associated with common DEGs. P-value < 0.01 was set as the threshold value.

### Establishment of the Prognostic Cox Model

The gene expression data (raw count) were normalized with the TMM method. We got the normalized counts per million mapped reads (CPM) value. A log-based transformation (log(cpm+1)) value was used for subsequent survival analysis. Firstly, we used univariate Cox regression analysis and the log-rank test to detect prognosis-related DEGs. The cutoff value for univariate Cox analysis was 0.20, and the cutoff P-value for the log-rank test was 0.10. To ensure the biological significance of the identified DEGs, filtered genes whose highest expression value (log(cpm+1)) among CN-AML and healthy donors less than 1.0 were removed. Then, the BeatAML dataset was used as the training cohort to construct the prognostic Cox model. Least absolute shrinkage and selection operator (LASSO) analysis and stepwise algorithm were applied simultaneously to select the most significant prognostic gene from the identified prognosis−related DEGs. The optimal values of the penalty parameter λ were determined through ten folds cross-validation. The optimal tuning parameter λ was identified via the min criterion. A prognostic Cox model was established based on a linear combination of the gene expression level multiplied by a regression coefficient (β). The risk score of the model was calculated as follows: risk score = expression of gene_1_ × β1 + expression of gene_2_ × β2 + … expression of gene_n_ × βn. We tested the proportional hazards assumption based on the scaled Schoenfeld residuals using the “survival” packages in the R platform.

### Validation of the Model

The risk score for each patient was calculated with constructed Cox model. Using the median of the risk score as the cutoff value, patients in each cohort were divided into high- and low-risk group. We applied a log-rank test to compare the overall survival difference between the high and low-risk group. Meanwhile, the time-dependent receiver operating characteristic (ROC) analysis was applied to calculate the area under the ROC curve (AUC) value at 1-, 2-, 3-years of the model. The AUC value of more than 0.5 indicates a non-random effect, and 1 indicating a perfect model (28). The GSE6891 included detailed genetic mutation information but no follow-up information. Therefore, these patients were classified into a favorable and adverse group assessed by ELN recommendations (6). The patient was defined to be favorable when FLT3-ITD is negative, and NPM1 is positive, or CEBPA double mutant is available. The patient was defined to be adverse if a sample has at least one of the following: (a) FLT3-ITD is positive and NPM1 is negative as well as CEBPA double mutant is not available. (b) EVI1 expression is positive. Risk scores were compared between two groups, and a Wilcoxon test P < 0.05 was considered statistically significant.

### Optimization of the Model With Three Independent Cohorts

Firstly, we enumerated all possible combinations of 12 genes included in the model. Specifically, we selected from 3 to 12 out of 12 genes to construct a new multivariate Cox model. We got 4017 combinations in total. Next, for each combination we constructed a new multivariate Cox model with selected genes in BeatAML. Then, for each combination, the new model was applied to predict risk scores for CN-AML patients in GSE12417, TARGET and BeatAML, respectively. We calculated the 1-, 2-, 3-years AUC value and the log-rank test’s P-value in these cohorts. Combinations filtering was executed based on the following criteria: 1) the minimum value of 1-, 2-, 3-years AUC value should more than 0.60 (28); 2) the maximum AUC of 1-, 2-, 3-years AUC value should more than 0.70; 3) the P-values from a log-rank test should less than 0.05 (The cutoff for TARGET was 0.10). Subsequently, we got the combinations that passed our filtering criteria. Then, we used a min-max normalization to scale the original ROC. Each ROC value was replaced according to the following formula.

$$Normalized\;AUC_{i}=\frac{\text{ AUCi}-\text{Min}\left(\text{AUC}\right)}{\text{Max}\left(\text{AUC}\right)-\text{Min}\left(\text{AUC}\right)}$$

We summed up all normalized AUC values in three independent cohorts in each combination and selected the combination with the largest AUC value. Finally, we constructed a new Cox model with the genes included in the combination with the largest AUC value.

### Comparison With Other Published Predictive Models for Prognostic Assessment

We screened publications from 2014 to 2020 on PubMed using the following keyword terms: (“CN-AML” OR “cytogenetical” OR “normal karyotype”) AND (“TCGA” OR “GEO” OR “biomarker” OR “prognosis” OR “prognostic”). We got three published models in total. The detailed model formulas were as follow: 1) MPG6 score = (0.0492 * CD52) - (0.0018 * CD96) + (0.0131 * EMP1) + (0.2058 * TSPAN2) + (0.0234 * STAB1) - (0.3658 * MBTPS1) (13); 2) 3-genes model = (0.2016 * ROBO2) + (0.1274 * IL1R2) - (0.5365 * SCNN1B) (14); 3) 7-genes model = (0.71900 * CD34) + (0.61927 * MIR155HG) + (0.67258 * RHOC) + (0.66929 * SCRN1) + (0.65925 * F2RL1) + (0.65777 * FAM92A1) + (0.61491 * VWA8) (29). We applied these models to four BMMCs datasets, which included BeatAML, TARGET, GSE12417 and GSE71014, to compare the performance of these models comprehensively.

### Statistical Analysis

In our study, overall survival (OS) was defined as the time interval between the date of diagnosis and the date of death or lost to follow-up. We conducted univariate Cox analysis, and factors with P-value <0.10 were incorporated into a multivariate Cox analysis, which was used to construct a prognostic Cox model and to identify independent prognostic factors. All statistical analyses were performed with the R 3.6.1 software (http://www.r-project.org/).

## Results

### Clinical Information and Dataset Quality Control

**Figure 1:**
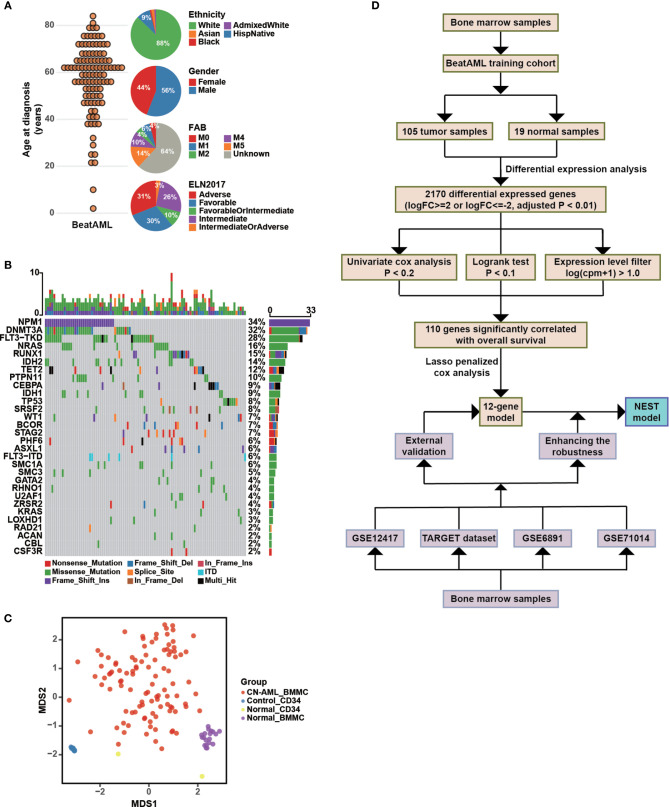
Clinical characteristics of CN-AML in the BeatAML cohort and analysis strategy. **(A)** The age distribution (left panel) and the clinical characteristics (right panel) of CN-AML patients in the BeatAML cohort. **(B)** Genetic mutation pattern in BeatAML CN-AML patients. **(C)** Multidimensional scaling (MDS) plot of all samples in the BeatAML dataset. **(D)** The overall flow chart of the bioinformatic analyses applied to this study.

We downloaded RNA-sequencing data and clinical information for *de novo* CN-AML patients from BeatAML (19), which included 105 BMMCs samples obtained from CN-AML patients (**Figure 1A**) and 33 samples from healthy donors (see *Methods*). The ages of the CN-AML patients ranged from 2 to 84 years, a large proportion of which were older than 40 years (88.57%). No significant difference in the sex composition was observed. According to the ELN recommendations, 30% of the patients in the BeatAML cohort had a good prognosis, 26% had an intermediate prognosis, and 31% had an adverse prognosis, which implied prognostic heterogeneity among the CN-AML population. The spectrum of genetic mutations in the BeatAML cohort was broad (**Figure 1B**), with 34.38% of the CN-AML patients harboring an *NPM1* mutation, which formed the largest subgroup, consistent with previous studies (6, 9, 30). Other common mutations included *DNMT3A* mutations (32.29%), *FLT3-TKD* mutations (28.12%), and *NRAS* mutations (15.62%). Among the 33 samples from healthy donors, 19 samples were BMMCs samples and 14 samples were bone marrow CD34^+^ cells. All of CD34^+^ cells were collected from three healthy donors. Notably, CD34^+^ sample from a single donor was included in each sequencing batch, and this sample (Control_CD34) was sequenced 12 times in total. Control_CD34 served as a quality check against intergroup batch effects. Few batch effects were observed for the BeatAML dataset (**Figure 1C**). We chose the 105 BMMC samples from CN-AML patients and the 19 BMMC samples from healthy donors in the BeatAML cohort for use in further downstream analyses. The overall flowchart used for the bioinformatics analysis is shown in **Figure 1D**.

### Association Between CN-AML Pathogenesis and Immune Dysfunction

**Figure 2:**
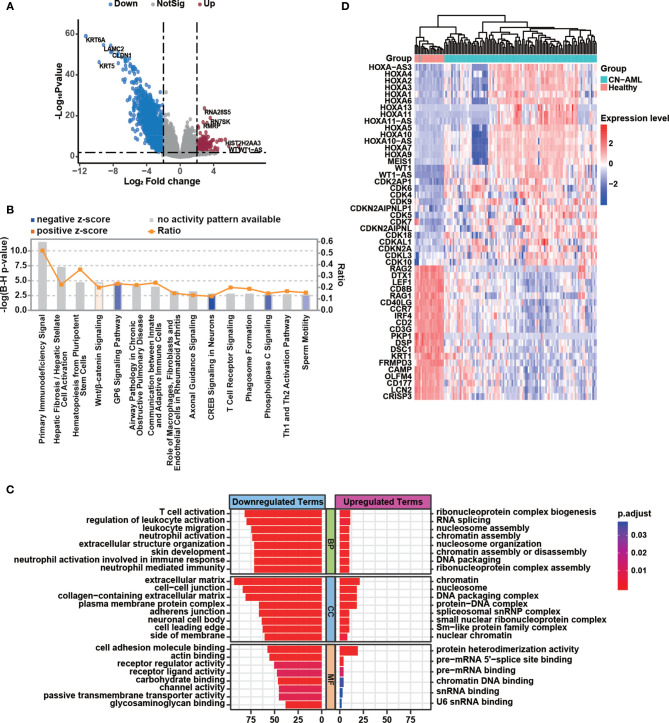
Immune dysfunction plays a vital role in CN-AML pathogenesis. **(A)** Volcano plot showing differentially expressed genes (DEGs) in bone marrow mononuclear cells (BMMCs) between CN-AML patients and healthy control. Upregulated genes in CN-AML are highlighted in red, and downregulated genes are highlighted in blue. **(B)** Canonical pathways enriched by Ingenuity Pathway Analysis (IPA) analysis. The orange bar indicates that the pathway in CN-AML is activated with a positive z-score. The blue bar indicates that the pathway is suppressed with a negative z-score. The gray bars indicate pathways for which no predictions can be made. **(C)** Enriched gene ontology (GO) terms for the upregulated and downregulated genes in CN-AML patients compared with healthy controls. **(D)** The expression of T cell and neutrophil activation-related genes and several well-known leukemia-related genes in CN-AML patients and healthy controls.

To identify differences in the BMMC transcriptomic profiles between CN-AML patients and healthy donors, we performed a differential gene expression analysis with edgeR, which resulted in the identification of 2,170 differential expressed genes (DEGs; **Table S2**), including 1,956 downregulated and 214 upregulated genes in CN-AML patients compared with healthy donors (**Figure 2A**). The identified DEGs included several known disease-linked genes, including cell cycle-related genes, HOX family genes (31), and *WT1* (32) (**Figure 2D**). To further explore the biological functions of the identified DEGs, we performed enrichment analyses. The IPA results suggested that the canonical Wnt/β-catenin pathway was activated in CN-AML, which agrees with previous reports (**Figure 2B**) (33, 34). Particularly, these identified DEGs were enriched in immune-related pathways, including primary immunodeficiency signaling, communications between innate and adaptive immune cells, and T cell receptor signaling. Furthermore, GO enrichment analysis revealed that the downregulated DEGs were primarily associated with the activation of immune cells, including neutrophils, leukocytes, and T cell (**Figure 2C**). We then examined the expression of several classical T cell and neutrophil activation-related genes in CN-AML (**Figure 2D**), which included *RAG2*, *IRF4* and *CD8*. The results revealed that these genes were significantly downregulated in CN-AML patients compared with healthy donors. These observations indicated that immune dysfunction was associated with CN-AML pathology.

### Prognostic Cox Model Construction

**Figure 3:**
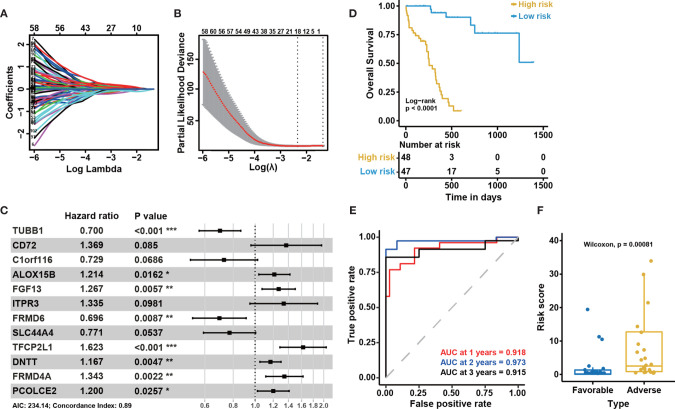
Construction of the 12-gene model and internal cohort validation. **(A)** LASSO coefficient profiles for the 110 prognosis-related differentially expressed genes. **(B)** Tenfold cross-validation for tuning parameter selection in the LASSO model. The solid vertical lines represent partial likelihood deviance ± standard error (SE) values. The dotted vertical lines are drawn at the optimal values according to the minimum criteria (left) and 1-SE criteria (right). **(C)** A forest plot showing the risk associated with gene expression for the genes included in the Cox model. Hazard ratio (HR) < 1 indicates that the gene is protective. Otherwise, it is a risk gene. P < 0.05 indicates that this gene is an independent prognostic factor (P-value significant codes: 0≤***<0.001≤**<0.01≤*<0.05). **(D)** Kaplan-Meier curves for overall survival based on the predicted risk score. The P-value for Kaplan-Meier curves is calculated by the log-rank test. **(E)** Time-dependent ROC curves for overall survival at 1, 2, and 3 years based on the 12-gene model. **(F)** The distribution of predicted risk scores in patients with favorable and adverse clinical outcomes, as assessed by European Leukemia Net (ELN) recommendations in the BeatAML cohort (n = 95).

To identify DEGs related to CN-AML prognosis, we performed univariate Cox and Kaplan-Meier (KM) analyses (see *Materials and Methods*). After the initial screening from all DEGs, we identified 110 DEGs significantly associated with the clinical outcome (**Table S3**). The prognostic impacts on AML of several of the genes we identified have previously been validated in previous studies, such as *CD72* (35), *ALOX12* (36), *CD7* (37), and *BMP2* (38). Using these 110 prognosis-related DEGs, we performed LASSO regression and stepwise regression analysis to confirm whether any combination of these DEGs could be used to accurately predict prognosis (**Figures 3A, B**). We identified 12 genes, which we used to construct a prognostic multivariate Cox model (**Figure 3C**). Because the proportional hazards assumption is critical to the Cox regression (39), we tested this assumption for our model. The proportional hazard assumption is supported by the finding of a non-significant relationship between residuals and time (40). And our results suggested that the test was not significant for all 12 genes, and the global test was also not statistically significant (**Figure S1**). Therefore, we could assume that the model met the proportional hazards assumption.

To confirm the association between these 12 genes and the clinical outcomes of CN-AML, we performed KM analyses for all 12 genes using the BeatAML cohort. We noticed that 10 of the 12 genes were significantly associated with prognosis (log-rank test P < 0.05, **Figure S2**). We then assessed the performance of the model, primarily focusing on two indicators: the P-value of the KM analysis (log-rank test) was used to evaluate a model’s ability to distinguish between patients with favorable and adverse prognoses, and the AUC value was used to evaluate the accuracy of the model. An AUC value above 0.5 indicates a non-random effect, with a value of 1 indicating a perfect model (28). In the KM analysis, low-risk patients had significantly improved overall survival (OS) compared with those in the high-risk group (log-rank test, P < 0.05, **Figure 3D**). The 1, 2, and 3-year AUC values for this model were 0.918, 0.973, and 0.915, respectively (**Figure 3E**). When we divided the CN-AML patients from the BeatAML cohort into favorable and adverse groups, based on ELN recommendations (6), the predicted risk score was able to clearly distinguish between the favorable and adverse groups (Wilcoxon test, P < 0.01, **Figure 3F**), which suggested that our model was generally consistent with clinical guidelines. All of these results implied that the 12-gene model could reliably predict the prognosis of CN-AML patients.

### External Validation of the 12-Gene Model in Four Independent Cohorts

**Figure 4:**
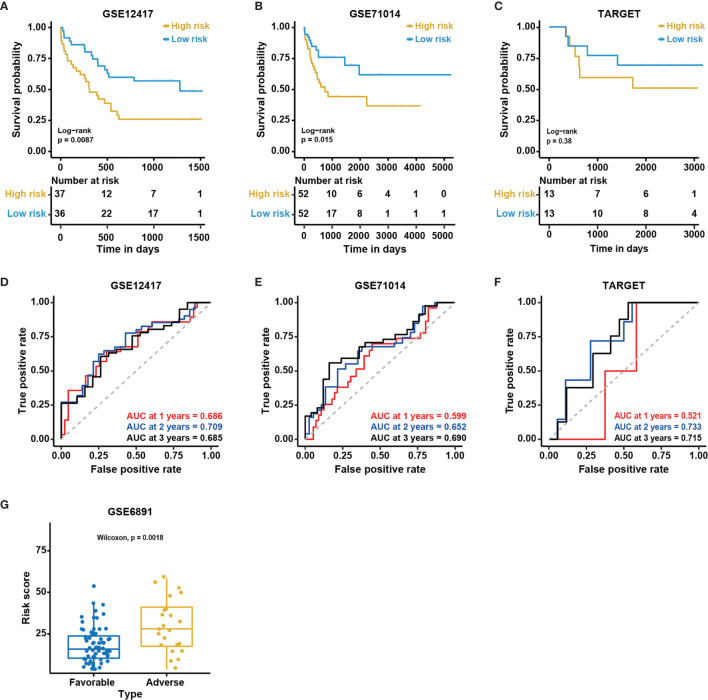
Validation of the 12-gene model in external cohorts. Kaplan-Meier curves for overall survival in different external independent cohorts, **(A)** GSE12417 (n = 73); **(B)** GSE71014 (n = 104); **(C)** TARGET (n = 26). The P-value for Kaplan-Meier curves is calculated by the log-rank test. Time-dependent ROC curves for overall survival at 1, 2 and 3 years in different external independent cohorts based on the 12-gene model, **(D)** GSE12417 (n = 73); **(E)** GSE71014 (n = 104); **(F)** TARGET (n = 26). **(G)** The distribution of predicted risk scores in patients with favorable and adverse clinical outcomes, assessed by European Leukemia Net (ELN) recommendations in GSE6891 (n = 88).

To further examine the performance of the 12-gene model, we applied the model to four external independent cohorts, including GSE12417 (n = 73), GSE71014 (n = 104), GSE6891 (n = 88), and TARGET (n = 26). The detailed demographic data for these cohorts are listed in **Table 1**. Similar to the outcome for the BeatAML cohort, the low-risk group had a significantly longer OS than the high-risk group for both the GSE12417 and GSE71014 cohorts (log-rank test, P < 0.05, **Figures 4A, B**). The AUC values at 1, 2, and 3 years for GSE12417 were 0.686, 0.709, and 0.685 (**Figure 4D**), and AUC values for GSE71014 were 0.599, 0.652, and 0.690 (**Figure 4E**). The AUC values for both the GSE12417 and GSE71014 cohorts approached 0.70, which suggested that the 12-gene model performed well in these two external independent cohorts. The survival analysis in the TARGET cohort indicated no significant difference between low- and high-risk groups (log-rank test, P > 0.05, **Figure 4C**). We speculated that the small cohort size and younger patients of the TARGET cohort contributed to this observation. Nevertheless, AUC values for the TARGET cohort at 1, 2, and 3 years were 0.521, 0.733, and 0.715, respectively (**Figure 4F**), which indicated that the model could be acceptable for the prediction of short-term clinical outcomes for pediatric patients. Moreover, we divided CN-AML patients from the GSE6891 cohort into favorable and adverse groups according to the ELN recommendations (see Methods). The predicted risk score was able to significantly distinguish favorable and adverse groups (Wilcoxon test P < 0.01, **Figure 4G**). The above results further validated the performance of the 12-genes model.

**Table 1:** Clinical characteristics of patients from multiple cohorts.

Characteristics	Bone marrow					Peripheral blood	
	BeatAML (n = 95)	GSE12417 (n = 73)	GSE71014 (n = 104)	GSE6891 (n = 88)	TARGET (n = 26)	BeatAML (n = 43)	TCGA (n = 60)
Age							
Median (yr)	60	62	NA	46	13	62	56
<60 yr	47 (49.5%)	31 (42.5%)	NA	83 (94.3%)	26 (100%)	18 (41.9%)	34 (56.7%)
>=60 yr	48 (50.5%)	42 (57.5%)	NA	5 (5.7%)	0	25 (58.1%)	26 (43.3%)
Sex							
Male	52 (54.7%)	NA	NA	45 (51.2%)	17 (65.4%)	23 (53.5%)	30 (50.0%)
Female	43 (45.3%)	NA	NA	43 (48.9%)	9 (34.6%)	20 (46.5%)	30 (50.0%)
FAB							
M0	4 (4.2%)	1 (1.4%)	NA	0	0	0	3 (5.0%)
M1	5 (5.3%)	21 (28.8%)	NA	23 (26.1%)	6 (23.1%)	1 (2.3%)	19 (31.7%)
M2	2 (2.1%)	33 (45.21%)	NA	12 (13.6%)	7 (26.9%)	1 (2.3%)	17 (28.33%)
M4	11 (11.6%)	9 (12.3%)	NA	18 (20. 5%)	6 (23.1%)	1 (2.3%)	11 (18.3%)
M5	15 (15.8%)	6 (8.2%)	NA	29 (33.0%)	3 (11.5%)	2 (4.7%)	9 (15.0%)
M6	0	3 (4.1%)	NA	2 (2.3%)	0	0	0
M7	0	0	NA	0	1 (3.9%)	0	1 (1.7%)
Unknown	58 (61.1%)	0	NA	4 (4.6%)	3 (11.5%)	38 (88.4%)	0
OS state							
Alive	56 (59.0%)	31 (42.5%)	68 (65.38%)	NA	16 (61.5%)	18 (41.9%)	18 (30%)
Death	39 (41.1%)	42 (57.5%)	36 (34.62%)	NA	10 (38.5%)	25 (58.1%)	42 (70%)

OS, overall survival; NA, not available.

### Enhancing the Robustness of the 12-Gene Model

**Figure 5:**
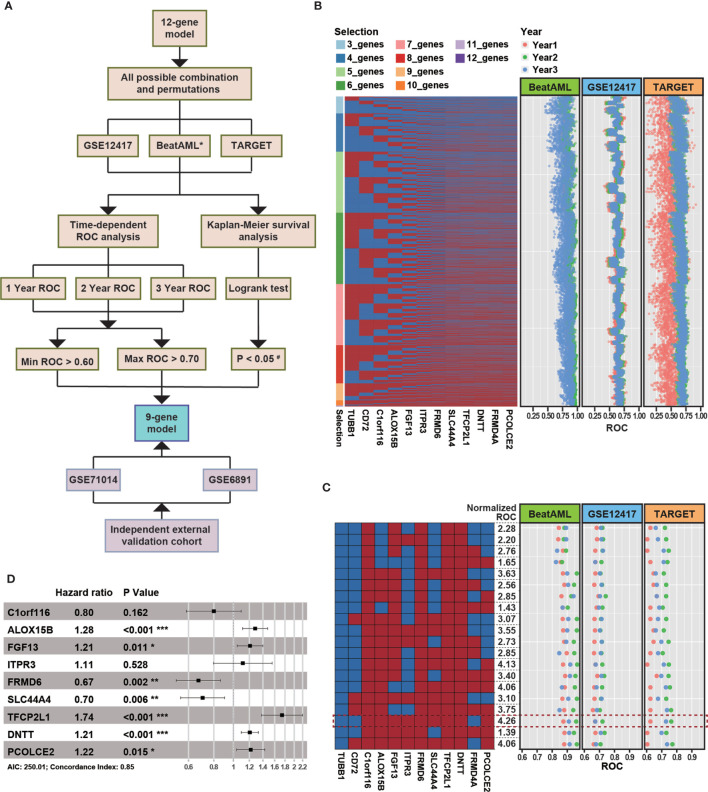
The strategy for enhancing the robustness of the model. **(A)** The overall flow chart for enhancing the robustness of the model. * indicates the cohort was used as a training dataset. The cutoff P-value for the log-rank test in the TARGET cohort was 0.10 (#). **(B)** The heatmap represents all combinations of 12 genes. Each column represents a gene, and each row represents a gene combination. In the heatmap, red rectangles denote selected genes, and blue rectangles denote unselected genes. The dot plot represents the area under the ROC curve (AUC) value for overall survival at 1 (red), 2 (green), and 3 years (blue) in various external independent cohorts based on the new model. **(C)** Combinations that passed the filtering criteria. The formula used to normalize the AUC can be found in Methods. The combinations highlighted with a red rectangle represent the combinate with the highest normalized AUC value. **(D)** A forest plot of the risk associated with the expression of each gene is included in the Cox model (P-value significant codes: 0≤***<0.001≤**<0.01≤*<0.05).

The 12 genes used in our model were determined by machine learning algorithms based only on the BeatAML cohort. Because we noted differences between the various cohorts, such as the age and sex distributions, we decided to optimize the model based on multiple cohorts simultaneously to improve the robustness of the model. The median age of the TARGET cohort was 13 years, which was quite different from those of the other examined cohorts. The distribution of FAB subtypes in the TARGET cohort also differed significantly from those in the BeatAML and GSE12417 cohorts. Moreover, the detailed demographic information for the GSE71014 cohort was unknown (**Table 1**). Therefore, we selected the GSE12417, TARGET, and BeatAML datasets to optimize the model, whereas GSE71014 functioned as an external validation dataset (**Figure 5A**). Specifically, we enumerated all possible combinations of the 12 identified genes, resulting in 4,017 total combinations (**Figure 5B**). We set strict criteria to filter the candidate combinations (see *Methods*). After filtering, we obtained 20 candidate combinations. We then calculated a normalized AUC value to determine the optimal combination (see *Methods*), and we selected the combination highlighted by the red box, which presented with the largest normalized AUC value (**Figure 5C**). Finally, based on nine selected genes, we developed a new Cox model (**Figure 5D**). The nine-gene model met the global assumptions of proportional hazards (**Figure S3**). We termed this nine-gene model NEST (Nine-gEne SignaTure).

**Figure 6:**
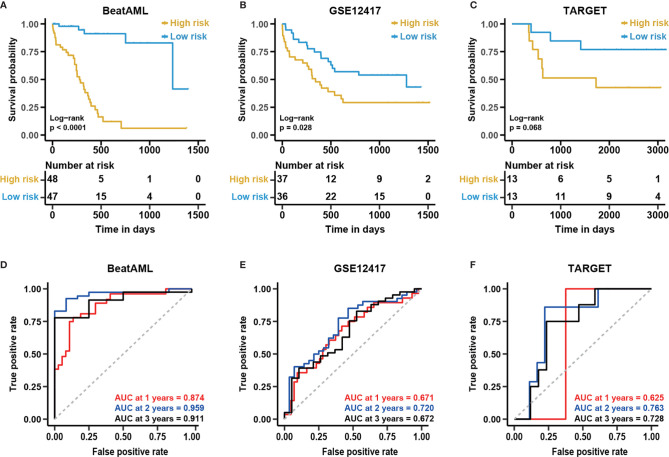
The enhanced performance of the NEST model in various cohorts. Kaplan-Meier curves for overall survival in different external independent cohorts, **(A)** BeatAML (n = 95); **(B)** GSE12417 (n = 73); **(C)** TARGET (n = 26). The P-value for the Kaplan-Meier curves was calculated by the log-rank test. Time-dependent ROC curves for overall survival at 1, 2, and 3 years in different external independent cohorts based on the 12-gene model, **(D)** BeatAML (n = 95); **(E)** GSE12417 (n = 73); **(F)** TARGET (n = 26).

**Figure 7:**
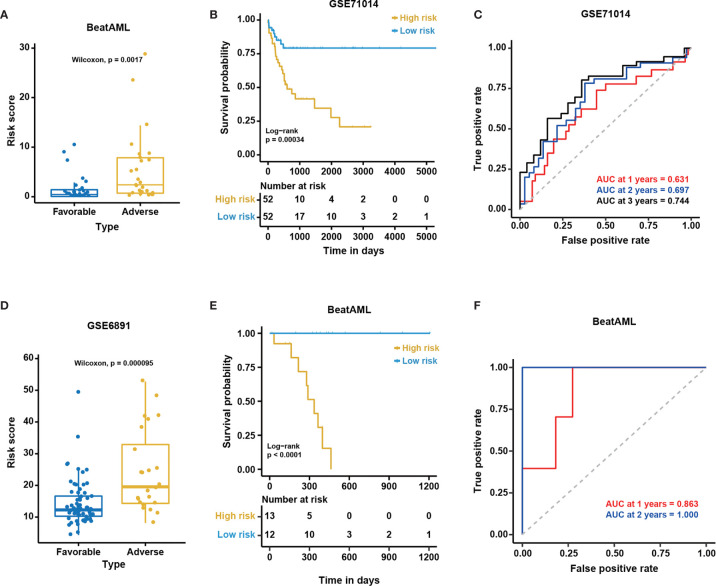
The excellent performance of the NEST model among external cohorts. The distribution of predicted risk scores among patients with favorable and adverse clinical outcomes as assessed by European Leukemia Net (ELN) recommendations in the **(A)** BeatAML and **(D)** GSE6891 cohorts. Kaplan-Meier curves for overall survival based on the predicted risk scores for individuals in the **(B)** GSE71014 (n = 104) and **(E)** BeatAML (n = 25) cohorts who were unable to be assessed by ELN. The P-value for Kaplan-Meier curves was calculated by the log-rank test. Time-dependent ROC curves for overall survival at 1, 2 and 3 years in **(C)** GSE71014 (n = 104) and **(F)** BeatAML (n = 25) patients who were unable to be assessed by ELN.

As shown in **Figures 6A, B**, the survival analysis inferred significant differences between the low- and high-risk group in the GSE12417 and BeatAML cohorts (log-rank test, P < 0.05). Although the log-rank test for the TARGET cohort was not significant, the performance of the NEST model was enhanced compared with that of the 12-gene model (**Figure 6C**). In addition, the AUC value for the BeatAML cohort slightly declined (**Figure 6D**), indicating no overfitting in the training data. The AUC value for NEST, when applied to GSE12417, appeared to be comparable to those obtained using the 12-gene model (**Figure 6E**). Notably, the AUC values for TARGET increased clearly (**Figure 6F**). According to the ELN recommendations, we divided the BeatAML cohort into favorable and adverse groups, and the predicted risk scores were able to significantly distinguish between these two groups (Wilcoxon test, P < 0.01, **Figure 7A**). These results indicated that the NEST model was more robust than the 12-gene model and performed well in both pediatric and adult CN-AML patients.

To further validate the generality of the NEST model, we used two additional external independent datasets, GSE71014 and GSE6891 (**Table 1**), to validate the model. The survival analysis showed significant differences between the low- and high-risk groups in the GSE71014 cohort (log-rank test, P < 0.05, **Figure 7B**). The AUC values for GSE71014 at 1, 2, and 3 years were 0.631, 0.697, and 0.744, respectively (**Figure 7C**), which was significantly enhanced compared with the 12-gene model. Additionally, the results in GSE6891 showed a high level of agreement with the ELN recommendations (Wilcoxon test, P < 0.01, **Figure 7D**). Because not every CN-AML patient harbors genetic mutations with prognostic significance (**Figure 1**), these CN-AML patients cannot be assessed by ELN guidance. Importantly, we were able to apply our model to these patients using nine gene expression levels to evaluate their prognosis. The performance of the NEST model for CN-AML patients who could not be assessed by ELN guidance was outstanding. The survival analysis inferred significant differences between the low- and high-risk groups (log-rank test, P < 0.05, **Figure 7E**), and the AUC values at 1 and 2 years were 0.863 and 1.000, respectively (**Figure 7F**). Even using fewer genes, these results indicated that the NEST model was more robust and performed better than the 12-gene model and worked well for patients who could not be assessed by ELN clinical guidance.

### Comparison of the NEST Model With Published Predictive Models for Prognostic Assessment

To further evaluate the performance of the NEST model, we compared our NEST model with other CN-AML prognostic models that were published from 2014 to 2020. These models included the MPG6 model (13), the 3-gene model (14), and the 7-gene model (29). We obtained each model’s formula from the corresponding literature (see *Methods*) and compared the performance using the BMMC datasets, including GSE12417, GSE71014, TARGET, and BeatAML. For these comparisons, we focused on two indicators: the P-value of the KM analysis (log-rank test), to evaluate each model’s ability to distinguish between patients with favorable and adverse prognoses, and the AUC value of each model, to reflect the accuracy. The AUC value of more than 0.5 indicates a non-random effect, and 1 indicating a perfect model.

The survival analysis showed that the risk score predicted by our model was significantly correlated with the survival of the patients in three out of four cohorts (log-rank test, P < 0.05, **Figure S4**). Although the P-value was higher than 0.05 for the TARGET cohort (log-rank test, P = 0.068), the difference between the high- and the low-risk group was clear. In contrast, other published predictive models could only distinguish between the low- and high-risk groups in at most two of the four cohorts (log-rank test, P > 0.05, **Figure S4**). The performance of the NEST model was stable in multiple cohorts, as reflected by the consistent high AUC values (**Table 2**). The MPG6 model exhibited excellent performance only for the TARGET cohort, which might suggest that this model is better suited for pediatric patients. The small size of the TARGET cohort may also account for this result. In the BeatAML and GSE12417 cohorts, the AUC values of the NEST model were consistently higher than those for the previously published models. At 1 year, although the AUC of the NEST model was lower than those for the MPG6 and 7-gene models for the GSE71014 cohort, the AUC values were higher than all models for the 2- and 3-year survival assessments. These results indicated that the performance of the NEST model was better and more robust than the performance of the other models across various cohorts.

**Table 2:** The AUC values of the ROC analyses in various cohorts using different predictive models.

		3-gene model	MPG6 model	7-gene model	NEST model
BeatAML	Year1 Year2 Year3	0.665 0.578 0.528	0.633 0.602 0.425	0.638 0.742 0.662	0.874 0.959 0.911
GSE12417	Year1 Year2 Year3	0.551 0.605 0.592	0.563 0.566 0.601	NA NA NA	0.671 0.720 0.672
TARGET	Year1 Year2 Year3	0.667 0.505 0.535	0.729 0.795 0.765	0.771 0.554 0.498	0.625 0.763 0.728
GSE71014	Year1 Year2 Year3	0.628 0.636 0.595	0.698 0.674 0.680	0.712 0.680 0.742	0.631 0.697 0.744

NA, not available.

### Independence of the Predicted Risk Score

**Figure 8:**
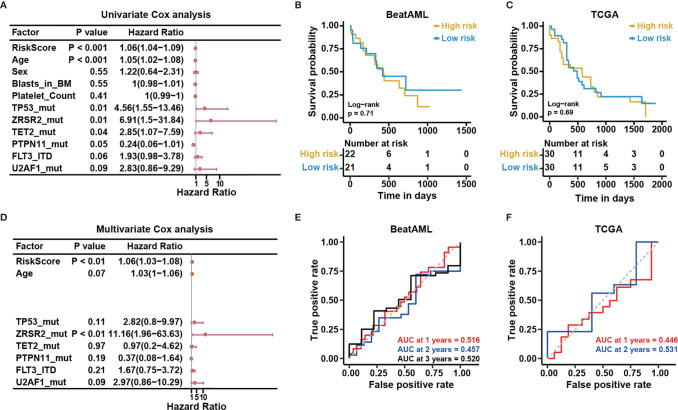
The risk score predicted based on bone marrow mononuclear cells is an independent risk factor. **(A)** Univariable Cox regression analysis of the relationship between the predicted risk score and common clinical outcomes. **(B)** Multivariable Cox regression analysis of the relationship between the significant factors in univariable Cox regression analysis (P < 0.10) and clinical outcomes. Kaplan-Meier curves for overall survival based on the predicted risk scores in the **(C)** BeatAML and **(E)** TCGA cohorts. The P-value for Kaplan-Meier curves was calculated by the log-rank test. Time-dependent ROC curves for overall survival at 1, 2, and 3 years in the **(D)** BeatAML and **(F)** TCGA cohorts.

Certain clinical characteristics and known genetic mutations could affect the prognosis of CN-AML patients; therefore, we next examined whether the risk score predicted by the NEST model could function as an independent prognostic factor that was not affected by other factors. First, we applied a univariate Cox analysis to common clinical factors and genetic mutations identified in the BeatAML cohort (**Table S4**). We found the NEST predicted risk score, age, *TP53* mutation, *ZRSR2* mutation, *TET2* mutation, *FLT3-ITD*, and *U2AF1* mutations were risk factors for a poor prognosis (**Figure 8A**), as reported by previous studies (6, 19, 41–44). In addition, the result suggested that *PTPN11* mutation was a protective factor for CN-AML, which appeared to contrast with previous reports (45). We believe that the low *PTPN11* mutation frequency among the BeatAML cohort could explain this discrepancy. Secondly, we selected those factors with P-values less than 0.1 in the univariate Cox analysis for inclusion in the multivariate Cox analysis. The result indicated that *ZRSR2* mutation was an independent risk factor (**Figure 8B**), which agreed with previous reports (42–44). Notably, the NEST predicted risk score was also an independent risk factor for poor clinical outcomes, which was not affected by age or the presence of other gene mutations. These results suggested that the NEST predicted risk score could serve as an independent prognostic factor in CN-AML.

### Applicability of the Model

Our reported results demonstrated the good results of the model among BMMC datasets. For clinical convenience, we next examined whether our model could apply to PBMC datasets. We selected the PBMC data (n = 43) from the BeatAML dataset. Meanwhile, we downloaded the TCGA-LAML (n = 151) from the TCGA data portal (https://gdc-portal.nci.nih.gov/), which is also a PBMC dataset. We selected CN-AML (n = 60) from the TCGA-LAML. Time-dependent ROC and KM analyses were applied to both datasets. No difference between the low- and high-risk groups was observed for either cohort (log-rank test, P > 0.05, **Figures 8C, D**). The AUC values were also not acceptable for these cohorts (**Figures 8E, F**). The above results illustrated that our model was only suitable for BMMCs, not for PBMCs, which implied subtle differences between PBMCs and BMMCs.

To further verify the differences between BMMCs and PBMCs in CN-AML patients, we first adjusted for confounding variables, including the percentage of blasts and tissue sources in the multivariate Cox analysis. We obtained the percentage of blasts in BMMCs and PBMCs from clinical records to perform the test. Moreover, we calculated risk scores for patients with the BMMC and PBMC samples. To correct for the effects of blast percentages and tissue sources, we included the risk scores and the percentages of blasts in the multivariate Cox analysis. The results suggested that the risk score predicted by our model was independent of the percentages of blasts and tissue sources (**Figure S5A**, P<0.01), indicating our previous results were driven primarily by the model rather than differences in the percentages of blasts and tissue sources. Furthermore, we found that the PBMC samples were more likely to cluster separately from the BMMC samples during unsupervised clustering when including all genes. As shown in **Figure S5B**, we divided all samples into three groups, named A, B, and C. To test the statistical significance of PBMC sample enrichment in these three groups, we performed a hypergeometric test. The results indicated that PBMC samples were not significantly enriched in group A (P = 0.907) and group B (P = 0.987). Notably, PBMC samples were significantly enriched in group C (P = 0.004). The unsupervised clustering results implied that PBMC samples were more likely to cluster separately from BMMC samples, which might account for the differences in the model application between these two populations.

## Discussion

Although specific genetic mutations have been associated with prognosis in CN-AML patients (6, 11, 46), the specific relationships between aberrant gene expression and clinical outcomes in CN-AML remain largely unknown. Novel biomarkers uncovered from transcriptome analysis that can provide prognosis assessment and potential targets for precision therapy strategies in CN-AML are urgently necessary. In this study, we integrated multiple cohorts to construct a multivariate Cox model, which we named the NEST model, to refine the risk stratification strategy in CN-AML patients.

The NEST model exhibited excellent robustness in five independent cohorts. The predictive capability of the NEST model for survival outcomes was validated by examining AUC values, which were greater than 0.70 in all cohorts (**Table 2**). Moreover, we also discovered that among CN-AML patients who could not be assessed by ELN recommendations, the performance of the NEST model remained outstanding (**Figures 7E, F**). However, the survival analysis of the NEST model in the TARGET cohort indicated no significant difference between the low- and high-risk group (log-rank test, P > 0.05, **Figure 6**). We believe that the small size of the TARGET cohort (n = 26) may explain this lack of significance. However, compared with the 12-gene model, the significance of the survival analysis and the AUC values were enhanced obviously by NEST for the TARGET cohort, which suggested that the NEST model was not only suitable for adult CN-AML patients but was also suitable for pediatric patients. Furthermore, the risk score predicted by the NEST model could function as an independent risk factor for CN-AML survival that was not affected by common clinical factors and genetic mutations (**Figure 8B**). Some limitations remain in this study that should be considered. In addition to the limited sizes of the CN-AML cohorts used to establish the NEST model, we only validated the NEST model on two external cohorts. Thus, the performance of the NEST model should be validated in further prospective studies to guide clinicians in the assessment of prognostic outcomes among CN-AML patients.

Despite these limitations, our NEST model showed more robust performance than three other models, which were published from 2014 to 2020, when tested in four independent cohorts (**Figure S5** and **Table 2**), which showed stable performance for both the survival and ROC analyses. In addition, our results revealed that the NEST model was only suitable for BMMCs, and could not be applied to PBMCs in CN-AML, indicating the existence of variability between BMMCs and PBMCs, which were not due to differences in the percentages of blasts (**Figure S5A**). The results of the unsupervised clustering further supported our conclusion (**Figure S5B**). Previous studies have provided insufficient evidence to support a lack of significant differences between BM and PB samples (21, 47, 48). Metzeler et al. (21) cited two pieces of literature (47, 48) to support the applicability of their model to both PB and BM samples. In the first cited study, Bullinger et al. (48) found that the expression profiles of three paired samples of PB and BM obtained from three patients were positively correlated according to unsupervised hierarchical cluster analysis. However, this result was not significant (n = 3), and this result could be interpreted as the patient heterogeneity was more significant than tissue source heterogeneity. In the second cited study, Sakhinia et al. (47) reported no significant differences in expression between BM and PB for 15 AML indicator genes. However, not only was the number of tested genes limited (n = 15) but also 5 of the 15 tested genes, representing one-third of the tested pool, showed significant differences. These findings argue against the interpretations represented by their conclusion. Moreover, differences have been found in the cell cycle phases between blasts from BM and PB (49–51), and recent studies have also indicated an increase in CD3^+^CD56^+^ T cells in the PB but not the BM of AML patients (52). Therefore, we believe that subtle differences do exist between PBMCs and BMMCs in CN-AML, and future studies should consider the sample origins more strictly.

Except for *ALOX15B* and *SLC44A4*, all of the genes included in our NEST model have previously been associated with leukemia [*FGF13* (53) and *DNTT* (54)] or other cancer types [*C1orf116* (55), *FRMD6* (56), *TFCP2L1* (57), *ITPR3* (58), and *PCOLCE2* (59)]. Princy et al. (55) found that *C1orf116* was associated with the epithelial to mesenchymal transition (EMT), which could represent a critical early event that occurs during tumor metastasis in multiple cancers. Furthermore, they demonstrated that the decreased expression of *C1orf116* was associated with poor prognosis in lung and prostate cancer patients, which is consistent with our results in CN-AML. *DNTT* has been reported to play important functional roles in VDJ recombination and T cell receptor (TCR) (60) and B cell receptor (BCR) (61) signaling, which might indicate an association between immune dysfunction and CN-AML pathogenesis. *FRMD6* has been associated with clinical outcomes in prostate cancer (56). Interestingly, *FRMD6* also plays a vital role in the Hippo pathway, which was originally identified as an evolutionarily conserved signaling pathway that controls organ size. An increasing amount of recent evidence has connected this pathway to the regulation of innate and adaptive immune responses (62–64). In addition, *TFCP2L1* has been reported to serve as a protective factor in clear cell renal cell carcinoma (57). However, our study suggested that *TFCP2L1* serves as a risk factor in CN-AML patients (**Figure 5D**), which could be explained by differences between tissue types. Notably, *TFCP2L1* has also been found to play an important role in stem cells as a component of a complicated transcriptional network that includes other key transcription factors, such as *Nanog*, *Oct4*, and *Sox2*, and maintains the pluripotency of mouse embryonic stem cells (mESCs) (65). Moreover, *TFCP2L1* is a downstream target of the leukemia inhibitory factor (LIF)/signal transducer and activator of transcription (STAT3) pathway, which mediates self-renewal (66). As a result, *TFCP2L1* might represent a potential target for anti-leukemogenic drug design.

Current dogma holds a “2-hit” model for leukemogenesis, which suggests that the development of AML is associated with dual dysfunction in cell proliferation and hematopoietic differentiation. Class I mutations, such as *FLT3-ITD* and *N-* or *K-RAS* mutations, confer a proliferative advantage to cells. Class II mutations serve primarily to block hematopoietic differentiation. As a result, aberrations in several canonical pathways associated with cell proliferation and differentiation, such as the STAT5, RAS/MAPK, PI3K/AKT, Notch, and Wnt pathways, have been associated with leukemogenesis (67). Given the particularity of cytogenetics in CN-AML, the specific leukemogenesis for CN-AML remains unclear. The current “2-hit” model only interprets the observed alterations that occur in blast cells. According to the NEST model, several immune cell-related genes may also be associated with CN-AML pathogenesis. In addition to *DNTT* and *FRMD6*, *ALOX15B* is constitutively expressed in human monocyte-derived macrophages. Although the function of *ALOX15B* in macrophages remains unclear (68), these immune-related genes suggest that immune dysfunction might also play a vital role in the pathogenesis of CN-AML. To summarize, we speculate that the development of CN-AML might be related to the dysfunction of immune cells in the BM microenvironment, which broadens our understand of the “2-hit” leukemogenesis model. However, more evidence remains necessary to confirm this idea in future studies.

In conclusion, this study identified nine prognosis-related genes in CN-AML and constructed an accurate and robust predictive Cox regression model that is suitable for BMMCs. The predicted risk score could serve as a powerful prognostic indicator, independent of other risk factors. Furthermore, our results shed new light on the pathogenesis of CN-AML and a new potential therapeutic target.

## Data Availability Statement

The original contributions presented in the study are included in the article/**Supplementary Material**. Further inquiries can be directed to the corresponding authors.

## Author Contributions

LY collected and analyzed the data and wrote the manuscript. LY and HZ interpreted the results. XY and TL edited the paper and revised the manuscript. SM, HC, KY, and TC revised the manuscript critically. KY and TC performed a final approval of the version to be published. All authors have read and approved the manuscript. All authors contributed to the article and approved the submitted version.

## Funding

This work was supported by grants from the Ministry of Science and Technology of China [2020YFE0203000], the National Natural Science Foundation of China [81890990, 81861148029].

## Conflict of Interest

The authors declare that the research was conducted in the absence of any commercial or financial relationships that could be construed as a potential conflict of interest.
