# Supplementary material for: Prognostic Prediction of Cytogenetically Normal Acute Myeloid Leukemia Based on a Gene Expression Model
Source: Front Oncol. 2021 May 27;11:659201. doi: 10.3389/fonc.2021.659201 (PMC8190396; doi:10.3389/fonc.2021.659201)
Supplement: Supplementary file 1 [file DataSheet_1.pdf]

Supplementary Material

Supplementary Figures

Figure S1

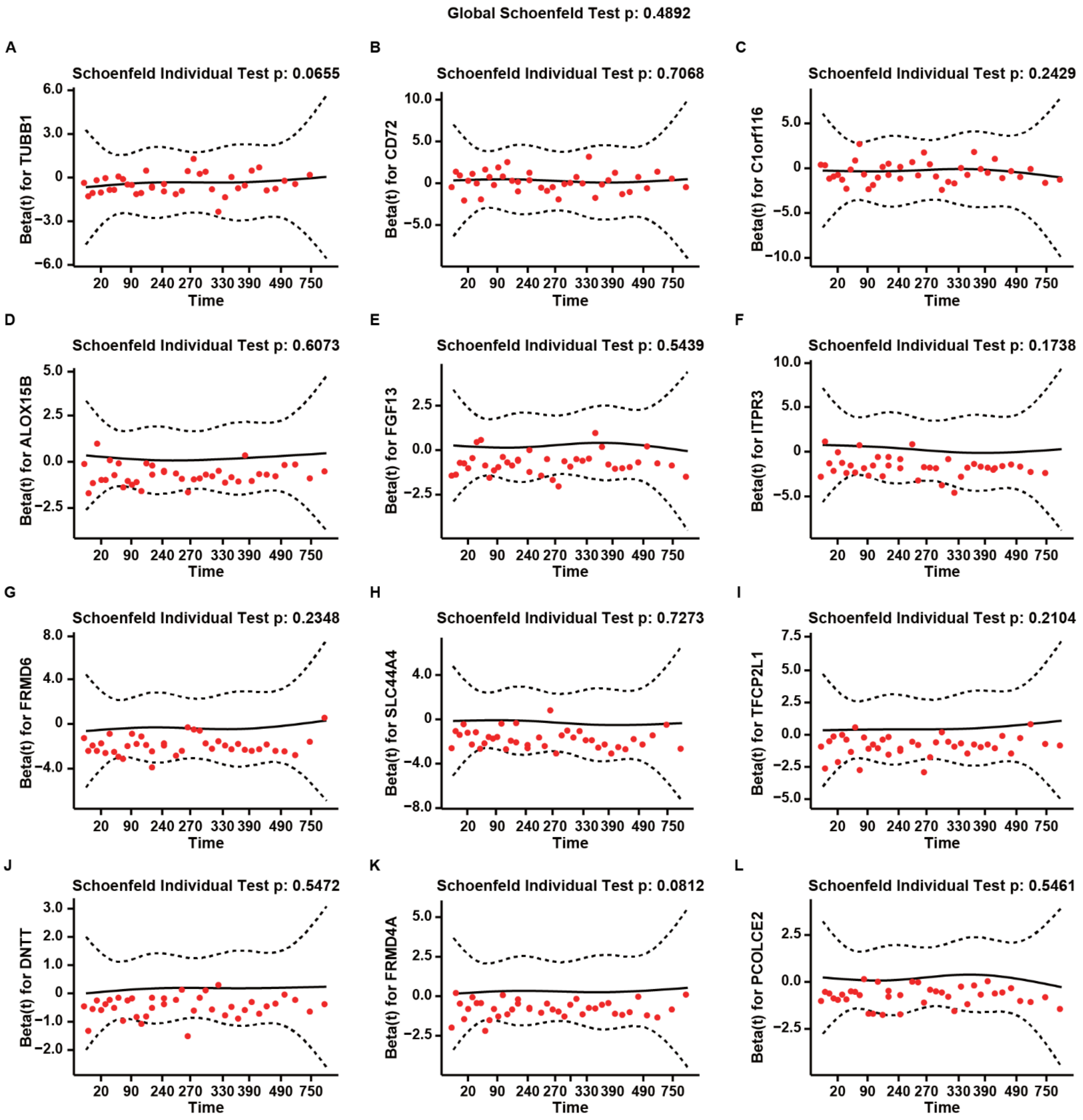

Figure S1. The 12-genes model meets the assumptions of a Cox model.

The plots of the Schoenfeld Residuals against the transformed time for model genes. According to the variation trend of the smooth curve and statistical analysis, it can be determined that there is no correlation between the partial residuals of four model genes and time rank ($P > 0.05$), suggesting that all model genes meet the assumptions of proportional hazards and suitable for Cox regression analysis.

Figure S2

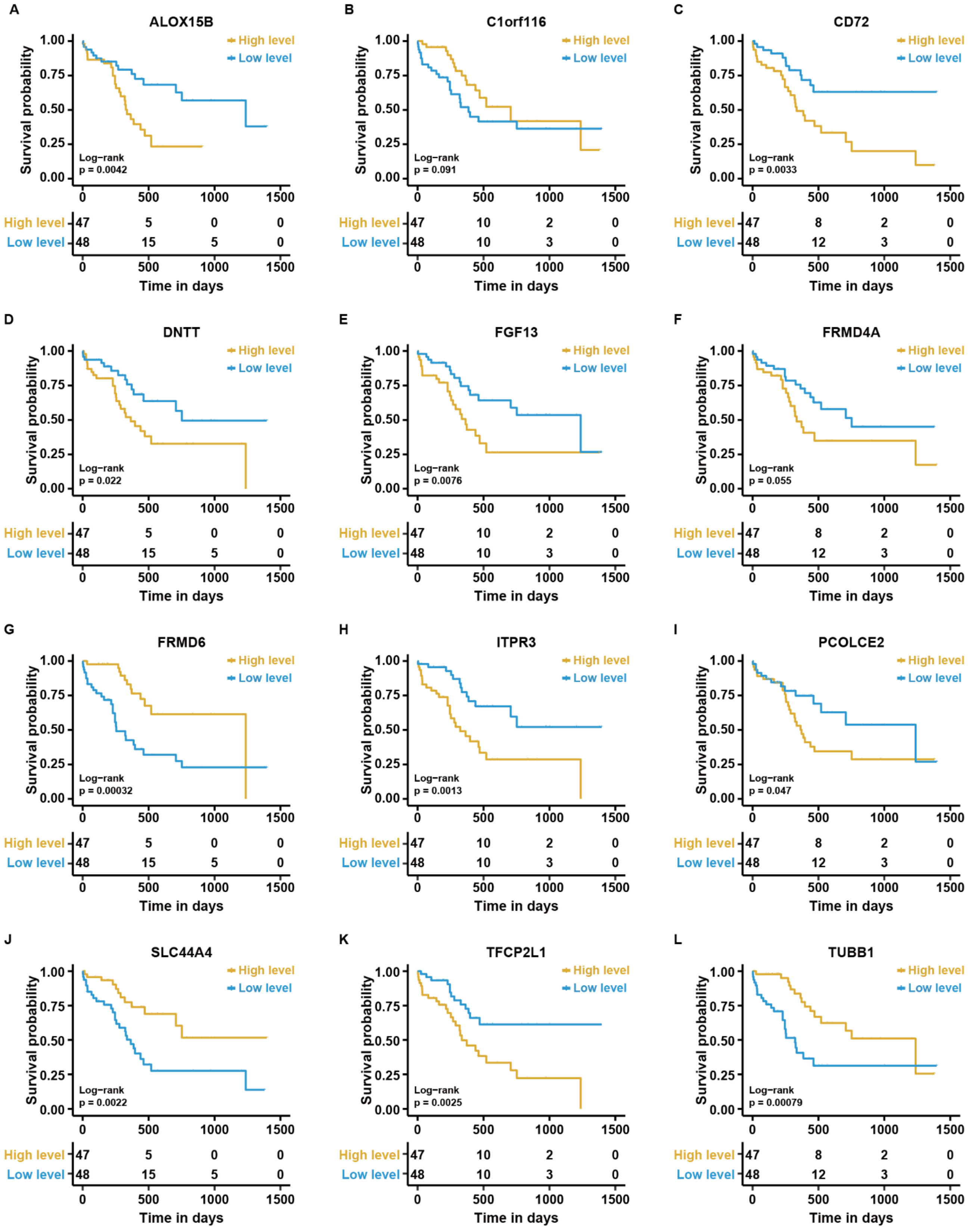

Figure S2. Genes in 12-genes model are associated with clinical survival outcomes.

The Kaplan–Meier curves of various genes for overall survival in BeatAML cohort, **(A)** ALOX15B; **(B)** C1orf116; **(C)** CD72; **(D)** DNTT; **(E)** FGF13; **(F)** FRMD4A; **(G)** FRMD6; **(H)** ITPR3; **(I)** PCOLCE2; **(J)** SLC44A4; **(K)** TFCP2L1; **(L)** TUBB1. The P value for Kaplan–Meier curves is calculated by log-rank test.

Figure S3

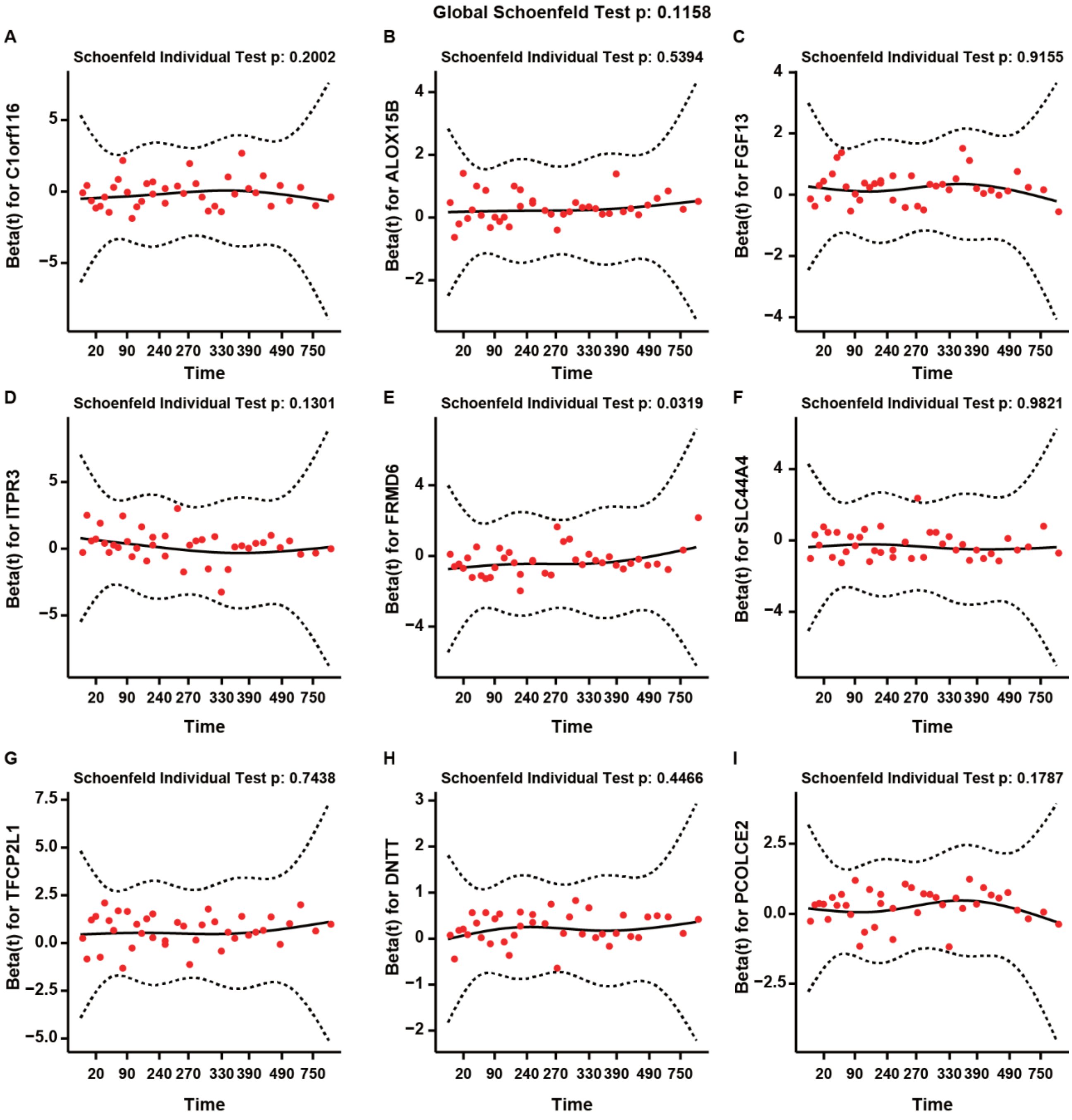

Figure S3. The NEST model meets the assumptions of a Cox model.

The plots of the Schoenfeld Residuals against the transformed time for model genes. According to the variation trend of the smooth curve and statistical analysis, it can be determined that there is no correlation between the partial residuals of four model genes and time rank (P > 0.05), suggesting that all model genes meet the assumptions of proportional hazards and suitable for Cox regression analysis.

Figure S4

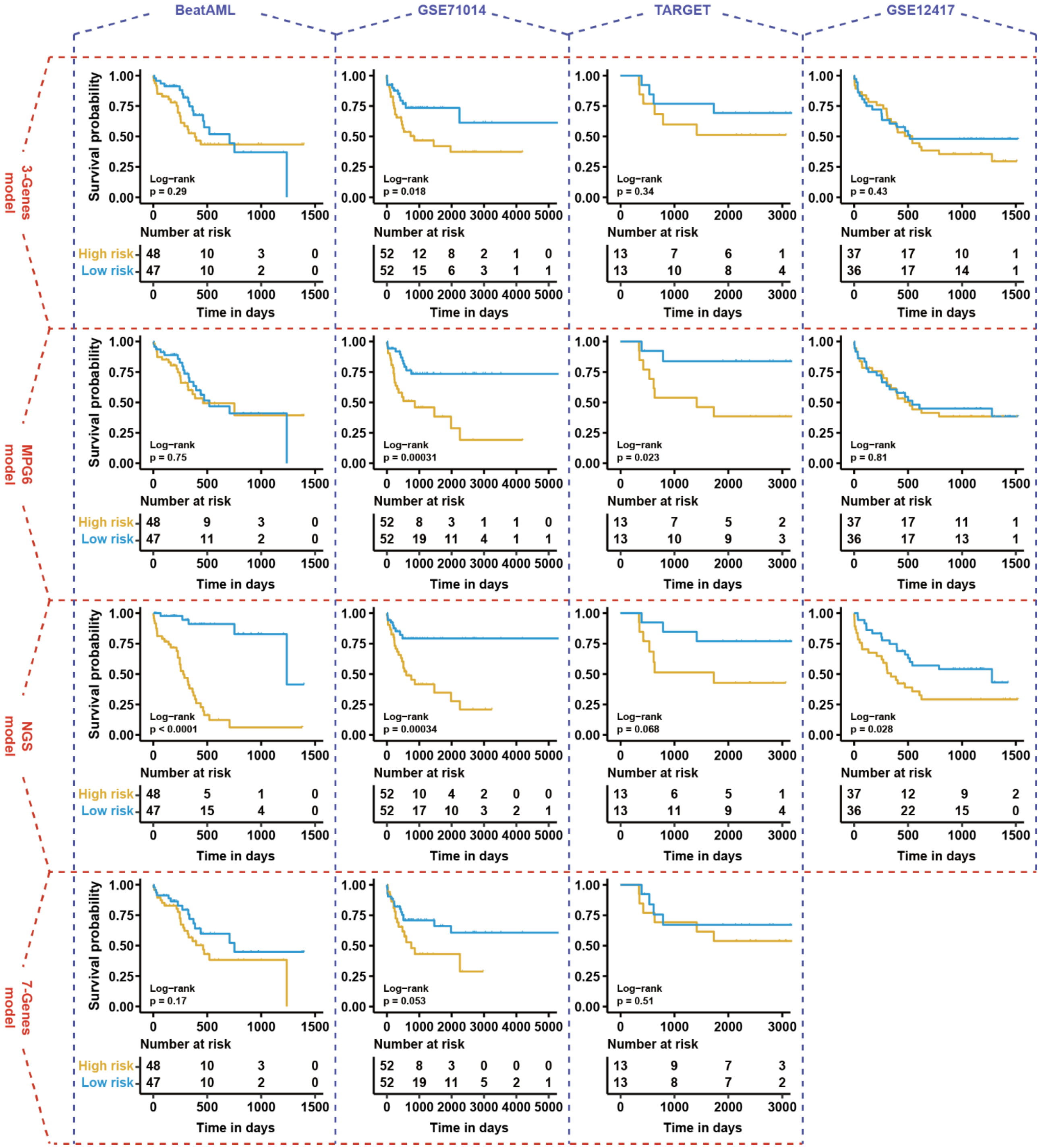

Figure S4. Comparison of NEST model with other published predictive models by survival analysis.

Kaplan–Meier curves for overall survival with four various models in four different external independent cohorts. Each row represents the performance of a model in various cohorts, and each

column represents the performance of various models in a cohort. The P-value for Kaplan–Meier curves is calculated by log-rank test.

Figure S5

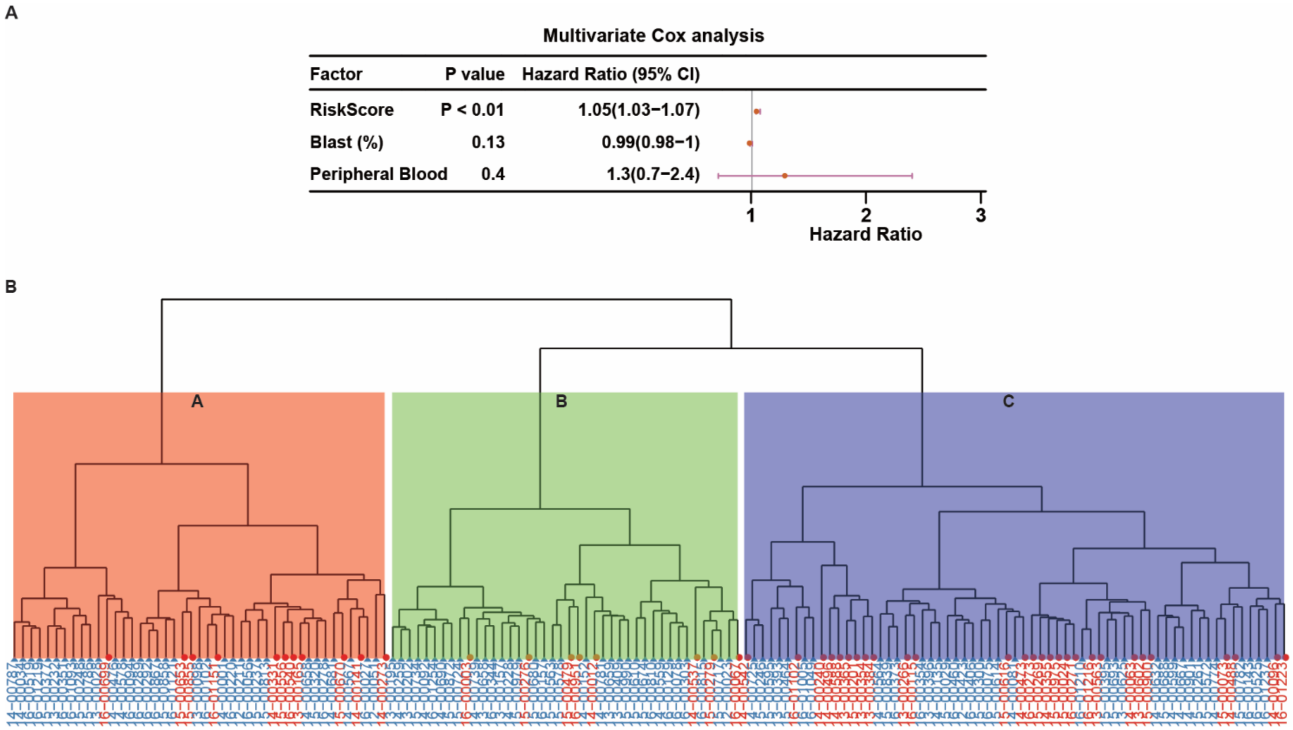

Figure S5. Differences exist between BMMCs and PBMCs in CN-AML patients.

(A) Multivariable Cox regression analysis of the relationship between factors and clinical outcomes. $P < 0.05$ indicates that the factor is an independent prognostic factor, not affected by the remaining factors in the analysis. **(B)** The unsupervised clustering of all CN-AML patients. All patients are divided into three groups: A, B, and C. The patient's name was shown on the x-axis. Names in red font represent PB samples, and blue font represents BM samples.
